# High and Low Contrast Visual Acuity Are Not Affected in Amyotrophic Lateral Sclerosis

**DOI:** 10.1371/journal.pone.0168714

**Published:** 2016-12-29

**Authors:** Heather E. Moss, Monica Samelson, Girish Mohan, Qin Li Jiang

**Affiliations:** 1 Department of Ophthalmology and Visual Sciences, Division of Neuro-ophthalmology, University of Illinois at Chicago, Chicago, Illinois, United States of America; 2 Department of Neurology and Rehabilitation, University of Illinois at Chicago, Chicago, Illinois, United States of America; The University of Melbourne, AUSTRALIA

## Abstract

The afferent visual system may be affected by neuro-degeneration in amyotrophic lateral sclerosis (ALS) based on observations of visual function impairment and retinal inclusions on histopathology in ALS patients. To test the hypothesis that visual acuity is impaired in ALS, we compared three measures of visual acuity in ALS patients (n = 25) attending a multidisciplinary ALS clinic and age matched control subjects (n = 25). Bilateral monocular and binocular visual acuities were assessed using high contrast (black letters on white background) and low contrast (2.5%, 1.25% grey letters on white background) visual acuity charts under controlled lighting conditions following refraction. Binocular summation was calculated as the difference between binocular and best monocular acuity scores. There were no associations between binocular or monocular high contrast visual acuity or low contrast visual acuity and amyotrophic lateral sclerosis diagnosis (generalized estimating equation models accounting for age). Binocular summation was similar in both amyotrophic lateral sclerosis and control subjects. There was a small magnitude association between increased duration of ALS symptoms and reduced 1.25% low contrast visual acuity. This study does not confirm prior observations of impaired visual acuity in patients with amyotrophic lateral sclerosis and does not support this particular measure of visual function for use in broad scale assessment of visual pathway involvement in ALS patients.

## Introduction

Clinical and post-mortem observations of pathological effects spreading beyond the motor system in some people with amyotrophic lateral sclerosis (ALS) have led to a shift from the classical characterization of ALS as a disease exclusively of motor neurons to that of a multisystem disorder.[1] Though disorders of the efferent visual system were first reported in the literature in 1978,[2,3] data to support afferent visual system involvement was not reported until twenty years later, when Munte et al described reduced visual evoked potentials in ALS patients.[4,5] Altered afferent visual function has also been documented using functional MRI,[6] and high and low contrast visual acuity (VA) measures, though these latter observations have not been confirmed.[7] Structural alterations in the retina may be the basis for these changes.[8–12] Reports of optic nerve involvement are conflicting.[9,13]

Psychophysical tests of visual function are based on subject response to visual stimuli and measure integrated neurological function of the afferent visual pathways. On the basis of reports of functional and structural changes in the afferent visual pathways of ALS patients, we hypothesize that psychophysical visual function tests are reduced in association with ALS. Visual acuity is a psychophysical test that measures central visual function. Low contrast VA (LCVA), tested using grey letters on a white background, has increased sensitivity for neurological visual pathway disease over high contrast VA (HCVA), which is tested using black letters on a white background.[14] HCVA and LCVA were chosen for this study based on a prior report demonstrating impairment in association with ALS, though this may have been confounded by non-standardized lighting conditions, lack of optimized refraction, and limitation to binocular measurements.[7] In the current study we sought to test the hypothesis that HCVA and LCVA are decreased in ALS subjects (i.e. confirm the prior observation), using gold standard VA testing methods. This objective was achieved.

## Methods

### Subjects

This is a cross-sectional study of a population of individuals with sporadic possible, probable or definite ALS according to the revised El Escorial criteria[15] attending a multidisciplinary ALS clinic serving the greater Chicago, Illinois, USA region. These clinical criteria were used due to their good sensitivity for prediction of pathological findings diagnostic of ALS.[16] Age matched (within 5 years) control subjects were recruited from healthy individuals accompanying those receiving care in the ALS multidisciplinary clinic and from the ophthalmology clinic. Participants were excluded if they had a history of neurological disease other than ALS or of ophthalmic disease other than refractive error. This study was carried out with the approval of the University of Illinois at Chicago Office for the Protection of Research Subjects. Written informed consent was obtained from all participants. Procedures followed were in accordance with the ethical standards of the responsible committee on human experimentation and with the Helsinki Declaration of 1975, as revised in 1983.

Age, gender, education, neurological, ophthalmic and medication histories were obtained for all subjects by self-report. ALS severity was assessed using the revised ALS functional rating scale including respiration (ALSFRS-R), a validated measure of symptom severity and functional limitation (range 0–48, 48 is normal)[17] and manual muscle testing (MMT), variations of which have demonstrated good reliability within and between raters and a more favorable coefficient of variation other than motor assessment methods (range 0–116, 0 is normal).[18]

### Visual acuity measures

A rigorous refraction and visual acuity (VA) measurement protocol based on that used in ophthalmologic and neurological trials was used.[19] Best spherical refraction for each eye on top of habitual corrective lenses was determined during monocular viewing of a high contrast chart (Sloan chart, Precision Vision, LaSalle, Illinois, USA) at 2m. For each viewing condition (monocular right eye, monocular left eye, binocular) VAs were assessed at 2m using one high (100%) and two low (2.5%, 1.25%) contrast charts (Sloan chart, Precision Vision, LaSalle, Illinois, USA) under standardized windowless examination room lighting. VA was recorded as number of characters correctly identified on each chart for each condition (range 0–60, 55 is 20/20 Snellen equivalent). Order of testing was randomized.

Inter-eye acuity difference, a potential marker of asymmetric disease effects, was calculated as the absolute difference between right and left eye VA measurements for a given contrast level. Binocular summation, a marker of post-geniculate visual processing, was calculated as the difference between binocular VA and best monocular VA for a given contrast level and considered to be evidence of summation if 5 or more letters were seen with both eyes than with the best eye, or inhibition if 5 or fewer letters were seen with both eyes than with the best eye.[20]

### Statistical analysis

A sample size of 25 ALS patients and 25 control subjects was selected to provide 86% and 84% power to detect the differences in HCVA and LCVA respectively, seen in our prior study of ALS and control patients[7] based on t-test for independent samples with alpha of 0.5 (PS power and sample size calculation version 3.0.43, Vanderbilt University[21]). This is likely an underestimate of the study power as we used generalized estimating equation models for comparison of monocular VA measures between groups. This is an efficient statistical method that considers multiple measures within subjects, accounts for their correlation and has increased power compared with comparison of single measurements from each subject.

Statistical analysis was performed using SPSS version 23 (IBM). Demographic variables were compared between ALS and control groups using independent samples t-test for continuous variables and chi square or Fisher exact test for categorical variables. Confounding effects of these on the relationships between VA outcome measures and ALS status were identified by including potential confounding variables of age and gender in the models and evaluating coefficient point estimates and their statistical significance. Interaction terms of these demographic variables and ALS diagnosis were also included to determine if the relationship varied depending on disease status. Variables demonstrating association with p ≤ 0.05 were included in models of VA outcome measures.

Monocular HCVA and LCVA were compared between ALS patients and control subjects using generalized estimating equation models accounting for within subject correlation. Binocular and inter-eye difference in HCVA and LCVA were compared between ALS patients and control subjects using linear regression models. Binocular summation and inhibition were compared between ALS patients and control subjects using contingency table analysis and logistic regression. In these models ALS status was treated as a dichotomous variable (i.e. ALS, not-ALS).

A second set of models used ALS function (ALSFRS-R, MMT) or duration as the independent variable of interest in order to examine for associations between ALS severity and monocular VA outcome measures. In these models it was assumed that control subjects had normal parameters (e.g. ALSFRS-R = 48, MMT = 0 and disease duration = 0).

## Results

Twenty-five ALS subjects and twenty-five age matched (within 5 years) control subjects were enrolled (Table 1). An additional four ALS subjects agreed to enroll, but were excluded for neurological co-morbidities (n = 1), ophthalmic co-morbidities (n = 1) or not meeting the ALS definition (n = 2). For one subject, ALSFRS-R and MMT were obtained on a day different than vision testing. All subjects except one ALS patient had at least a high school education. Medication information was not available for 4 ALS subjects. No subject with medication information available used a medication that has been reported to influence visual acuity.

**Table 1:** Subject characteristics.

	ALS (n = 25)	Control (n = 25)	P*
Age (mean (range))	52.9 yrs (26–77)	53.3 yrs (35–72)	0.92 (t-test, t = 0.099)
Gender (female)	32% (8)	64% (16)	0.09 (Fisher’s exact)
Corrective lenses distance	36% (9)	68% (17)	0.15 (Fisher’s exact)
Corrective lenses near	52% (13)	72% (18)	0.56 (Fisher’s exact)
Additional refractive correction**	52% (13)	68% (17)	0.53 (Fisher’s exact)
ALSFRS-R (mean, range)	37.25 (15–48)		
Symptom duration (mean, range))	2.5 yrs (0.2–7.7)		
MMT (mean, range)	21.5 (0–73)		

*t-test for continuous variables, chi square or fisher exact test for dichotomous variables

** correction provided in addition to habitual lenses following refraction protocol

Models to assess confounding by demographic variables identified age to be inversely associated with all VA measures and gender to not be associated with any VA measure at the p ≤ 0.05 level. The magnitude of the associations between age, gender and VA measures were similar for ALS and control patients (i.e. interaction terms were not significant in the models). Therefore age was included in all subsequent models of VA outcomes.

**Fig 1:**
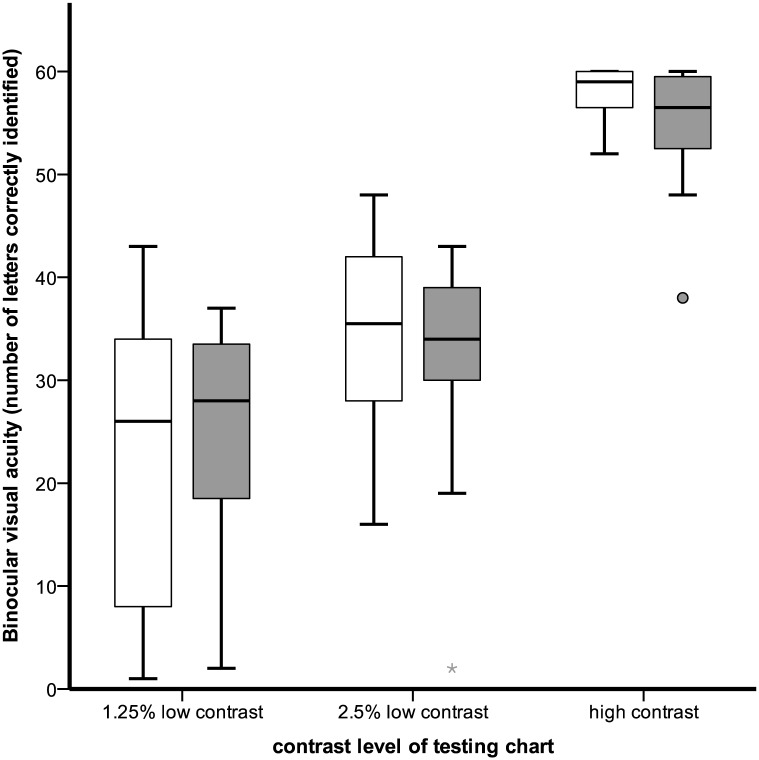
Binocular visual acuity by contrast level and ALS status. Gray boxes show ALS subjects and white bars show control subjects with similar high and low contrast acuity to ALS subjects. Boxes represent 25^th^-75^th^ percentiles. Error bars represent 5^th^-95^th^ percentile. Single markers represent outliers.

Binocular VA for varying levels of contrast among ALS and control subjects is shown in Fig 1. In linear regression models accounting for age, neither binocular HCVA, 2.5% LCVA nor 1.25% LCVA were associated with ALS (HCVA: coefficient point estimate -2.04 95% CI[-4.03,0.27], p = 0.08; 2.5% LCVA: -2.83[-6.98,1.31], p = 0.18; 1.25% LCVA: 0.49{-5.75,6.72, p = 0.876). In GEE models accounting for age, age*ALS interaction and within subject correlation between eyes, ALS was not associated with a difference in monocular HCVA (-2.61[-15.32,10.10], p = 0.69), 2.5% LCVA (-0.74[-19.3,17.9], p = 0.94) or 1.25% LCVA (7.05{-15.93,30.03], p = 0.55).

Inter-eye HCVA and LCVA differences were similar between ALS and control subjects (HCVA 0.88[-1.56,3.33], p = 0.47, 2.5% LCVA -0.20[-3.00,2.60], p = 0.89, 1.25% LCVA 0.60[-1.73,2.92], p = 0.61, linear regression accounting for age). Binocular summation prevalence was similar between groups with 3 control and 1 ALS subjects having HCVA binocular summation (p = 0.40, Fisher’s exact), 13 control and 11 ALS having 2.5% LCVA binocular summation (exp(B) 1.21[0.36,4.04], p = 0.76, logistic regression accounting for age) and 11 control and 13 ALS having 1.25% LCVA binocular summation (exp(B) 0.56[0.16,1.94], p = 0.36, logistic regression accounting for age). Binocular inhibition was rare, occurring with HC testing in 1 control subject, 2.5% LC testing in 1 ALS subject and 1 control subject and not at all during 1.25% LC testing.

Coefficient estimates from GEE models of monocular high and low contrast VA representing ALS as a continuous variable based on neurological function and disease duration variables, with control subjects assumed to have normal neurological function and disease duration of zero, are summarized in Table 2. Disease duration had a statistically significant (p = 0.01) coefficient estimate for 1.25% LCVA with a point estimate corresponding to 1.92 fewer letters seen for each additional year since ALS symptom onset. Functional impairment as measured by ALSFRS-R had a borderline significant association (p = 0.07) with coefficient point estimate corresponding to 0.3 fewer letters seen for each point increase in ALSFRS-R. Neither 2.5% nor 1.25% LCVA binocular summation were associated with ALSFRS-R (p = 0.77, 0.92), MMT (p = 0.11, 0.78) or disease duration (p = 0.68, 0.23) (logistic regression accounting for age).

**Table 2:** Regression model coefficient point estimates (95% CI) for models of VA outcomes as a function of ALS disease variables with control subjects assumed to have no limitations.

Visual acuity outcome	Functional impairment (48-ALSFRS-R)	Functional impairment (MMT)	Disease duration (years)
100% HCVA	-0.10 (-0.32, 0.11)	-0.01 (-0.09, 0.10)	0.24 (-0.74, 1.22)
	p = 0.35	p = 0.93	p = 0.63
2.5% LCVA	-0.15 (-0.50, 0.21)	-0.04 (-0.21, 0.17)	-0.71 (-2.95, 1.53)
	p = 0.41	p = 0.68	p = 0.53
1.25% LCVA	-0.30 (-0.61, 0.02)	-0.04 (-0.19, 0.12)	-1.92 (-3.42, -0.42)
	p = 0.07	p = 0.64	p = 0.01

HCVA: high contrast visual acuity, LCVA: low contrast visual acuity, ALSFRS-R: amyotrophic lateral sclerosis functional rating score with respiration, MMT: manual motor testing,

Coefficients are from generalized estimating models of monocular VA accounting for age and within subject correlation

## Discussion

This cross-sectional study of high and low contrast visual acuity in ALS subjects does not confirm our previous observation of decreased binocular visual acuity in ALS subjects. Possible reasons for this include unmeasured confounding variables in the prior study such as variable exam room lighting and lack or refractive correction. Though both studies were completed in ALS referral centers with similar demographics, it is possible that there are unmeasured variables that differed between the two studies leading to disparate results. We also did not find differences in monocular HCVA or monocular LCVA and therefore we reject the hypothesis that VA is decreased in association with ALS diagnosis. We did not find evidence for laterality in visual pathway dysfunction, as is typically the case with motor manifestations of ALS. We also did not find evidence of higher order visual pathway impairment impairing binocular summation or causing binocular inhibition. Our results do not support VA as a diagnostic marker of ALS. Strengths of this study include the use of rigorous visual acuity measurements, including refractive correction and an age matched control group. Limitations include a sample size not powered for subgroup analyses and the possibility of type II error. Though ALS and control groups were not matched for gender, this was not identified as a confounding variable in our analysis.

In a secondary analysis that represented ALS as a continuous variable based on either neurological function or disease duration, we did find evidence of association between 1.25% monocular LCVA duration of ALS symptoms at the p = 0.01 level and evidence of association between 1.25% monocular LCVA and ALS related neurological dysfunction as measured with the ALSFRS-R at the p = 0.07 level. These results counter those in our prior study, which did not show an association between VA and other markers of ALS disease progression. However, they build on a study demonstrating association between a retinal structural measure and pulmonary function tests in ALS.[12] While this is an intriguing result with regard to understanding involvement of the visual system in ALS, the relevance to disease monitoring or patient disability is likely minimal as the point estimates of these effects are small (-1.92 letters per year of symptoms and -0.3 letters per lost point on ALSFRS-R), which are not within the range of clinical meaningfulness and are within the test-rest margin of error.[22]

Literature demonstrating an association between decreased high and low contrast visual acuity and ALS motivated this (non)confirmatory study of visual acuity in ALS patients.[7] The results can not be extrapolated to other visual function tests as each of these captures unique aspects of afferent visual function. In fact, the structural abnormalities of the retina that have been reported in ALS patients are unlikely to affect visual acuity. Tests of color discrimination or contrast sensitivity may be more sensitive to disruptions in ganglion cells or the inner retina. These have been reported to be normal on average in a small sample (n = 12) of ALS patients, though one third of subjects had color discrimination falling in the abnormal range.[9] Further research is needed to confirm these findings and to determine if there is an effect of ALS severity.

An important area of future research is the structure-function relationship in the afferent visual pathway in ALS patients. A single case demonstrated mild pre-morbid contrast sensitivity impairment in a patient subsequently found to have retinal inclusions on post mortem examination.[11] Unfortunately, the remainder of the literature, including this study, is limited to reports of structure or function.

## Conclusion

Previous research by our group found decreased high and low contrast visual acuity in ALS patients compared with control subjects. In this study, which used a more rigorous visual acuity measurement protocol, we did not confirm this result. However a secondary analysis found associations at the p<0.01 and p = 0.07 level between 1.25% low contrast visual acuity and ALS duration and functional impairment. While the results do not support development of visual acuity as a clinical marker of ALS disease, they do support further research into visual pathway involvement in ALS.

## Supporting Information

S1 Data High and low contrast visual acuity measurements in ALS and control subjects raw data. Separate sheets in the file contain raw data, data formatted for statistical program input for linear regression, GEE, and visualization.
(XLSX)
